# Effectiveness analysis of papillary thyroid carcinoma (PTC) diagnostics in Holycross Cancer Centre (HCC) in Kielce according to fine needle aspiration biopsy quality assurance program in pathology department between years 2001 and 2012

**DOI:** 10.1186/1756-6614-6-S2-A59

**Published:** 2013-04-05

**Authors:** Jacek Sygut, Aldona Kowalska, Janusz Słuszniak, Jacek Heciak, Janusz Kopczyński, Dominik Sygut, Kornelia Niemyska, Stanisław Góźdź

**Affiliations:** 1 Pathology Department, Holycross Cancer Centre in Kielce, Kielce, Poland; 2 Endocrinology Department, Holycross Cancer Centre in Kielce, Kielce, Poland; 3 Oncological Surgery Department, Holycross Cancer Centre in Kielce, Kielce, Poland; 4 Radiology and Imaging Diagnostics Department, Holycross Cancer Centre in Kielce, Kielce, Poland; 5 Pathology Department, Medical University of Lodz, Lodz, Poland; 6 Holycross Cancer Centre Board, Kielce, Poland

## Introduction

Commonly accepted evaluation of diagnostic test usefulness on the basis of analysis of such factors as sensitivity, specificity and overall accuracy is a prelude to more profound statistic deliberations. Practical meaning of those factors depends on many elements like representativity of FNAB smear and also final microscopic evaluation, tumour type or diameter.

## Aim of the study

To evaluate detection of PTC by ultrasound-guided fine needle aspiration biopsy (USG-FNAB).

## Material

Between years 2001 and 2012, a group of 26361 patients had thyroid lesions diagnosed by USG-FNAB. At least one carcinoma was diagnosed (including two metastases) in 343 patients. Surgical specimens (SS) revealed 419 malignancies, with 335 PTCs. A diameter of histologically measured PTCs ranged from 0.5 mm to 170mm, where those correctly diagnosed in USG-FNAB was between 3 and 170 mm. Number of false negative (FN) cases ranged from 0 to 16 each year.

## Methods

A correlation was sought between mean diameter of PTSc, FN cytologic cases and whole number of PTCs found in surgical specimens, each year, in analyzed period of time.

## Results

On the basis of numerical and proportional results of assessed parameters three periods of time can be distinguished in analyzed 12 years. The first one is between years 2001 and 2002. More PTCs with greater diameter were diagnosed with USG-FNAB and the percentage of FN diagnoses was higher than in the rest of the whole analyzed period. Since year 2008 till 2012 the number of FN diagnoses stabilized and reached the percentage value interval between 0 and 5. Strong negative correlation was statistically shown between the percentage of FN diagnoses and elapsed time (R= -0.7591, p=0.0042) as well as between the mean PTC diameter (R= -0.6011, p=0.387) and elapsed time.

## Conclusions

Presented data showed that, in the course of time, smaller diameter of PTCs was diagnosed by USG-FNAB and more rarely false diagnoses were given in Holycross Cancer Centre.

